# Assessment of Gait Patterns during Crutch Assisted Gait through Spatial and Temporal Analysis

**DOI:** 10.3390/s24113555

**Published:** 2024-05-31

**Authors:** Marien Narvaez Dorado, Miguel Salazar, Joan Aranda

**Affiliations:** Department of Automatic Control, Universitat Politècnica de Catalunya, Barcelona Tech—UPC, Edifici Omega, Campus Nord Jordi Girona, 1-3, 08034 Barcelona, Spain; miguel.antonio.salazar@upc.edu (M.S.); joan.aranda@upc.edu (J.A.)

**Keywords:** assisted gait, assistive devices, gait patterns, gait analysis, instrumented crutches

## Abstract

The use of crutches is a common method of assisting people during recovery from musculoskeletal injuries in the lower limbs. There are several different ways to walk with crutches depending on the patient’s needs. The structure of crutch gaits or crutch gait patterns varies based on the delay between the aid and foot placement, the number of concurrent points of contact, and laterality. In a rehabilitation process, the prescribed pattern may differ according to the injury, the treatment and the individual’s condition. Clinicians may improve diagnosis, assessment, training, and treatment by monitoring and analyzing gait patterns. This study aimed to assess and characterize four crutch walking patterns using spatial and temporal parameters obtained from the instrumented crutches. For this purpose, 27 healthy users performed four different gait patterns over multiple trials. Each trial was recorded using a portable system integrated into the crutches, which measured force, position, and acceleration. Based on the data angle, an algorithm was developed to segment the trials into gait cycles and identify gait phases. The next step was to determine the most appropriate metrics to describe each gait pattern. Several metrics were used to analyze the collected data, including force, acceleration, angle, and stride time. Among 27 participants, significant differences were found between crutch gait patterns. Through the use of these spatial and temporal parameters, promising results were obtained for monitoring assisted gait with crutches. Furthermore, the results demonstrated the possibility of using instrumented crutches as a clinical tool.

## 1. Introduction

Walking is a critical factor for independence, physical activity, and well-being. According to the World Health Organization (WHO), over a billion people worldwide required assistive technologies (e.g., wheelchairs, communication aids, crutches, canes, etc.) in 2019. These devices are fundamental to assisting elderly or disabled people in daily living, social participation, and quality of life. In the context of ambulatory assistive devices, forearm crutches have been used as assistive walking devices for a broad demographic. Various disabilities are assisted by crutches, ranging from short-term use such as ankle sprains, or lower limb surgery for a permanent loss of mobility due to amputation or Multiple Sclerosis [1,2].

Individuals with neurological mobility disorders and the elderly often rely on walking aids consistently. Forearm crutches facilitate partial and non-bearing gait, enhancing balance, stability, muscle activation, propulsion, and potentially alleviating walking-related pain [3,4]. Despite their evident benefits, prolonged and improper use of crutches is associated with secondary injuries and complications. These injuries include joint pain, crutch palsy, aneurysms, and repetitive use injuries [1,5,6,7]. Understanding the quantitative parameters involved in crutch gait is essential for clinicians and researchers to optimize patient care, ensuring appropriate gait training and crutch fitting [8].

### Walking with Crutches

Rehabilitation outcomes are often evaluated subjectively, relying on various standards and scales, potentially limiting patient recovery assessments. Integrating sensor-based systems for gait analysis provides an objective and reliable approach to monitoring, identifying, and quantifying gait parameters. In this context, instrumented crutches offer a cost-effective, noninvasive, and portable solution. Previous studies have proposed instrumented crutches to monitor several gait variables, such as axial forces on the crutch, crutch positions, upper limb forces [9,10], accelerations in the three-dimensional planes [8], ground reaction forces [11], joint angles and gait phases [12,13], step length [14], etc. For example, in [8] a system was developed to provide clinicians with quantitative parameters of upper limb contributions during walking with crutches. Similarly, in [15] the tip of a crutch was instrumented to measure axial forces and motion to detect the crutches’ different gait phases. These studies have reported different dynamic and kinematic aspects of crutch locomotion.

Crutch-assisted walking patterns are influenced by factors such as the delay between the placement of the ambulatory device and the foot, the number of simultaneous contact points, and laterality, as indicated by [6]. Distinguishing between these patterns remains a significant challenge and crucial aspect in customizing therapy to meet patients’ individual needs. Ref. [16] further extends this understanding by predicting various crutch-assisted gait patterns. These models could allow physiotherapists to choose the optimal crutch walking pattern for a specific subject and contribute to the design and control of exoskeletons, particularly in scenarios necessitating crutch support for equilibrium maintenance. Additionally, recent investigations into the spatio-temporal aspects of gait, as highlighted in studies such as [17,18], offer valuable insights for evaluating crutch gait performance. These insights hold the potential for tailoring treatments and selecting the most appropriate crutch gait pattern.

Despite qualitative descriptions of crutch walking patterns in clinical rehabilitation, a comprehensive set of metrics can enable a more precise evaluation of patient gait. This study proposes that crutch gait walking patterns can be objectively described and assessed using common spatial and temporal gait parameters. Our data collection methodology involved the instrumentation of forearm crutches to capture forces, accelerations, and angles within a diverse cohort of healthy individuals. Subsequent data processing included the development of an algorithm designed to analyze gait data and track the evolution of parameters across the gait cycle. This approach facilitated the segmentation of gait into individual strides for each user within each pattern, enabling a thorough analysis. The paper is structured as follows: Section 2 details the experiments and data collection process, Section 3 outlines the data processing steps and the methodology for segmenting gait patterns, Section 4 presents the findings and a comparative analysis of the gait patterns, and finally, Section 5 and Section 6 summarize and discuss the key outcomes of this study.

## 2. Materials and Methods

### 2.1. Participants and Experiments

The study followed the guidelines of the ethics committee of the Universitat Politecnica de Catalonia, the San Joan de Deu Hospital and the Helsinki Declaration. Participants were eligible for the experiments based on the following criteria:

- Age >18 years old;
- Ability to walk without assistance;
- No lower-extremity injuries or any gait pathology;
- Provide informed consent to participate.

**Figure 1:**
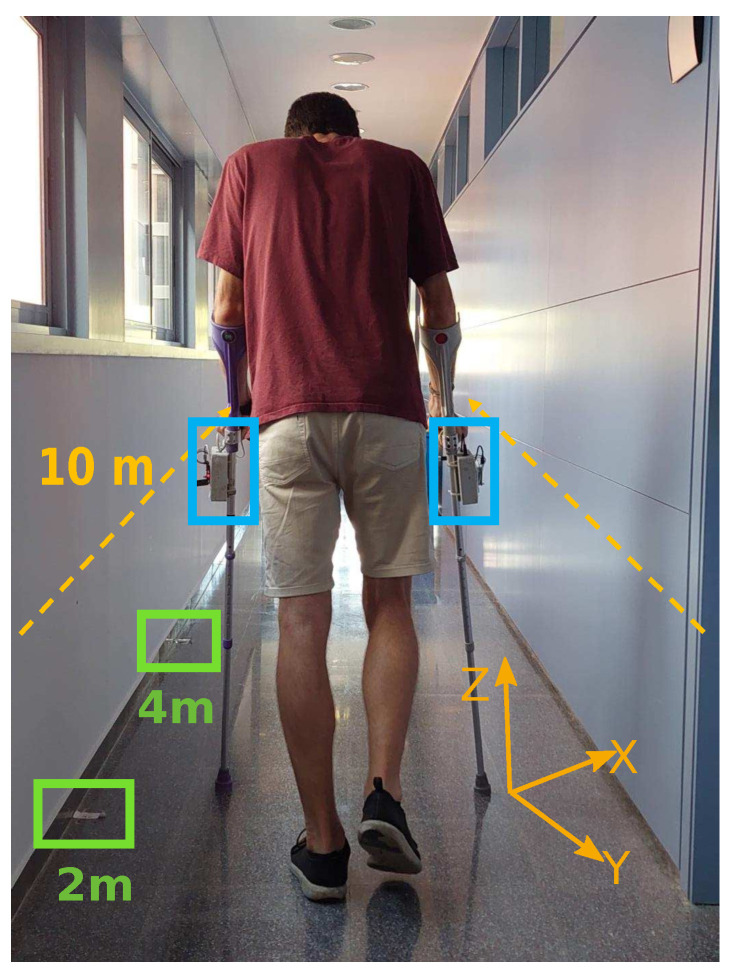
Subject walking with the instrumented crutches along the 10 m corridor performing a three-point gait. Green squares represent markers located every 2 m. Blue squares indicate the circuit boxes for each crutch.

The exclusion criteria included an evident pathology or disorder of overall coordination, which would affect gait when using crutches. Therefore, a sample of thirty-three healthy individuals was chosen using a non-probabilistic convenience sampling method. This approach was chosen for practical reasons, considering the feasibility and convenience of recruiting participants for the study. Six participants did not complete the experiments. The sample consisted of 27 healthy people, with a mean age of 27.81 years (SD = 10.15), with a minimum of 18 years and a maximum age of 60 years (16 men and 17 women). All the volunteers signed a consent form following the Helsinki Declaration. Details about the participants may be found in Appendix A. It was decided to select four gait patterns based on the general conventions used to refer to ambulatory assisted gait [19] and the literature review. These patterns are two-point (2P), three-point (3P), four-point, and swing-through. Participants received guidance on the proper usage of both forearm crutches, including walking techniques and handling instructions. During the session, they viewed instructional videos, led by physiotherapists, demonstrating the prescribed patterns. Researchers provided additional support, correcting any potential errors in execution. The team continuously accompanied participants throughout the process, adhering to the protocols outlined in the Oxford University Hospitals NHS Trust Guide [20], which dictated the sequence of presented patterns. Each subject conducted three trials for every gait pattern. In each trial, the participants walked 10 m (10 m, Walk-Test) back and forth, and the velocity was at a free cadence. Each volunteer traveled 20 m per trial, completing 12 trials in total. Throughout the trajectory, marks were placed every two meters on the floor to guide the participants. Figure 1 shows a participant walking along a 10 m corridor.

#### Instrumented Crutches

Each crutch contains force sensors and an inertial measurement unit (IMU). To detect axial forces, eight strain gauges were integrated into the handle of the crutches. Instrumentation amplifiers were used to amplify signals. An IMU sensor with three axes of acceleration, a three-axis gyroscope, and a three-axis magnetometer was utilized to measure the crutch pitch angles. Angles and accelerations estimated by the IMU are based on the reference system shown in Figure 1. The processing unit (Arduino NANO) was responsible for conditioning, converting, and transmitting the acquired data. Real-time data were sent via Bluetooth and stored on a standard PC for subsequent processing. In each crutch, data were sent at a frequency of 75 Hz. The system for each crutch was powered by a 5V/800 mAh power bank, allowing two weeks of daily use before recharging.

### 2.2. Data Collection

Data were collected using the same configuration for every trial for each participant, as shown in Figure 1. Every trial began with the participant standing in the initial position marked on the floor. After the volunteer was ready, each crutch was manually restarted using a switch and the data acquisition program started collecting. The system automatically calibrated within five seconds. During the user’s walk, the crutches sent data in real-time via Bluetooth to the computer for analysis and processing. After the completion of each trial, the data were saved and the system was restarted to begin collecting new data.

#### Parameters

In light of past research and collaborative ventures with therapists, we developed instrumented crutches. These crutches aim to capture linear axial forces, crutch angles, and accelerations in all three axes. Additional details about the prototype utilized for this purpose can be found in [21]. The relevance of these parameters is described as follows:

- Force: During the body swing phase of crutch walking, the upper limbs transfer ground reaction forces (GRF) to the upper limbs. Consequently, the percentage of applied body weight (BW) indicates whether crutches are used correctly or incorrectly. Furthermore, crutch gait requires greater forces on the upper and lower extremities, which causes increased energy expenditures [6].
- Angle: Angles provide information about the range of motion of the crutch and gait phases. Earlier research has also shown the relation between the position of the crutch and the stability during crutch walking [6].
- Linear Accelerations: Previous studies have shown that this parameter is related to gait stability. In stable walking, acceleration occurs on a periodical basis, while in an unstable gait, acceleration occurs aperiodically [6].

## 3. Data Processing

The crutch gait data collected were segmented into cycles to ease their analysis. Since both crutches captured the cyclic behavior of the gait, it was necessary to define a “dominant” crutch whose signals were used to detect the events that separate the trials into different gait cycles. The convention for this study was to consider the right crutch as the dominant one. Therefore, the gait cycles were defined by the data comprised between two consecutive initial events, detected using the right crutch. The obtained gait sequences for each pattern are presented in our previous work [21]. At each trial, every measure was recorded for each user along the trajectory. The duration of every cycle was normalized to analyze the signals between 0% and 100% of the gait cycle. Force data were normalized for each user according to their weight. The data set was categorized by user, gait pattern, trial, side and signal type (force, angles, accelerations).

### 3.1. Gait Cycle Segmentation

After data processing, crutch movements (pitch angles) were used to identify gait phases and segment the data into cycles, as conducted in previous work [21]. We identified two phases during the crutch movement: swing and stance. During the swing phase, the user moves the crutch along a trajectory in the air in the direction of walking. The stance phase, on the other hand, is characterized by the continuous contact of the crutch tip with the ground, which causes the crutch to follow an inverted pendulum motion.

Two main events divide these phases: crutch stance start and crutch swing start. They indicate the gait instants at which the crutch initiates contact with the ground and the instants at which the crutch stops this ground contact, respectively. We observed how at the swing start events, the pitch angle signal presents a negative peak. On the other hand, stance start events were detected by obtaining the time instants at which the pitch angle signal showed a positive peak.

These events were detected by a heuristic-based algorithm developed for this purpose, as expressed in the Algorithm 1.
**Algorithm 1** Heuristic-based algorithm for pitch events detection (left/right crutch)slope_thval_thtime_th**while** *k* < n° of samples **do**▹Evaluate every pitch signal sample    c1_sw = val_th > pitch(k)▹Pitch value condition swing event    c1_st = val_th < pitch(k)▹Pitch value condition stance event    c2_sw = time_th < time(k) - time(prev_sw)▹Pitch value condition swing event    c2_st = time_th < time(k) - time(prev_st)▹Pitch value condition stance event    c3_sw = slope_th < pitch(k) - pitch(k-1)▹Slope condition swing event    c3_st = −slope_th > pitch(k) - pitch(k-1)▹Slope condition stance event    **if** c1_sw AND c2_sw AND c3_sw **then**        swing_event = k ▹Swing event detected        Add detected event to vector of swing events    **else if** c1_st AND c2_st AND c3_st **then**        stance_event = k ▹Stance event detected        Add detected event to vector of stance events    **end if****end while**Swing events filtering▹Remove consecutive swing eventsStance events filtering▹Remove consecutive stance events

Three conditions are evaluated for each pitch signal sample to detect a maximum or minimum peak.

- Condition 1: Pitch value threshold. A swing start event occurs when the pitch signal reflects a pitch value lower than a threshold. Also, stance start events are considered if the pitch value of the sample exceeds a threshold.
- Condition 2: Time threshold. A swing or stance event is defined as a sample of the pitch signal detected after a specific time period has passed. This condition is implemented to enable the detection of only one event per peak.
- Condition 3: Slope threshold. Pitch signal samples are considered swing start events if their slope is positive and higher than the threshold for previous samples. In addition, it is considered a stance start event if the slope relative to the previous sample is negative and lower than a threshold. This condition allows us to detect the slope change corresponding to a peak.

**Figure 2:**
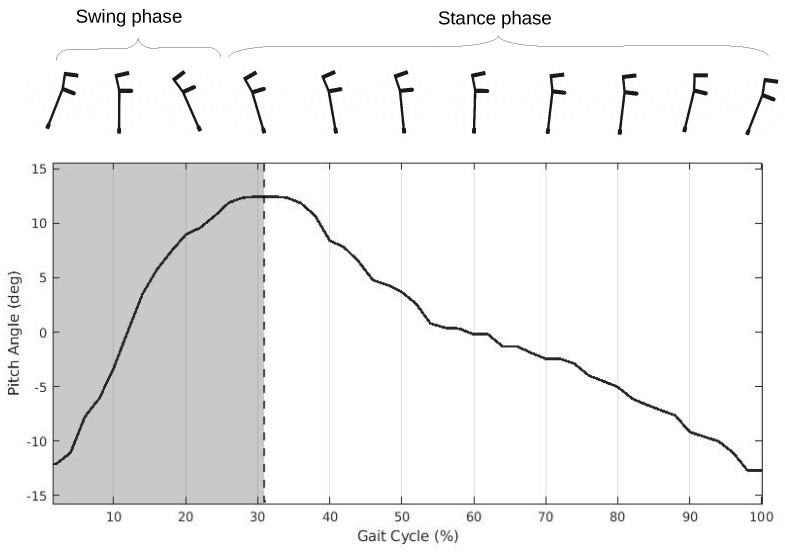
Crutch gait cycle division according to pitch angle peaks.

Some users had unusual gait movements (starting or ending cycles, half-turn movements, poor performance, etc.), leading to the detection of consecutive swing start events (with no stance start events in between). After applying the three conditions outlined above, the events that were wrongly detected were filtered. This filtering allowed the posterior segmentation of the crutch cycles, defining them as the periods between two swing start events, with the stance start event dividing the gait phases. This gait division is illustrated in Figure 2, taking a 2P gait signal as an example for visualization.

The number of cycles varies depending on the user and the walking pattern, going from 10 to 30 cycles in each trial for the collected data. In the signal processing process, it was noticed that the crutch’s linear acceleration signals were very sensitive to noise, external disturbances, or random movements of the user. To enhance the quality of the data, all the cycles that did not belong to a gait pattern were filtered out during the data processing.

### 3.2. Spatial and Temporal Parameters

For each gait pattern, spatial and temporal parameters were calculated to analyze how the person interacts with the crutches. By segmenting a gait trial into phases, it was possible to identify the different cycles occurring within it. It is, therefore, necessary to decide what information to extract from these cycles and to develop methods to extract it accurately. A set of metrics of interest was proposed based on force, accelerations, pitch and time variables. The parameters of interest for this project were divided into three main categories as shown in Table 1.

For each user, a dataset containing all their data was extracted. In [21] we identified a set of parameters to classify and describe four gait patterns during assisted crutch walking using different Machine Learning Techniques (ML). The extracted features were analyzed using feature selection methods and their relationship to various gait patterns. According to our findings, maximum force (left and right), maximum pitch angle (left and right), and stride time are the most accurate features. These parameters will be discussed further in this paper. A stride time is measured as the elapsed time between a swing event and the following swing event of one crutch. Furthermore, swing and stance phase parameters will be presented for a better understanding of the segmentation process. These measurements represent the percentage of the gait cycle corresponding to the swing and stance movements of the crutch along a single cycle.

## 4. Results

In this section, the results of the analysis of the database containing all the users are presented. As mentioned before, the database is organized by patterns which contain the trials and the gait cycles for both sides. Every cycle contains forces, angles, accelerations, and stride time, as well as the segmentation into swing and stance phases. To demonstrate the repeatability of the gait patterns, we will first present some statistical parameters for all users. Then, to observe the characteristic profile of the patterns, the graphics will be compared with plots for one particular user. The graphics show data for the right and left sides to analyze laterality.

### 4.1. Descriptive Statistics

Spatial and temporal variables for the left and right crutches for each of the four walking patterns are shown in Table 2 and Table 3). Table 2 summarizes the mean value of the maximum and minimum values of each parameter for every gait pattern. Mean and standard deviation (SD) were calculated for all subjects for all the cycles and trials. The primary analysis provides insights into individuals or groups of participants. For example, the maximum and minimum values can help identify extreme values or outliers that may be important to consider in subsequent analyses. The SD can provide information about the spread of the data and the degree of variation across the different cycles. Force measurements were normalized as a percentage of the body weight (BW). Maximum values of the forces show significant differences between them for both sides, but the values for each pattern remain within a range. Furthermore, forces have the highest SD of all measurements. Based on the comparison of measured forces the highest forces were applied by the user when performing patterns at three points and swing-through. During these types of gaits, the crutch advances simultaneously with the injured leg. Swing-through, on the other hand, is performed by the crutch user landing and pivoting over the crutch while lifting one leg (the impaired leg). Consequently, the person must apply more force to go forward with crutches in this pattern compared to others, 83%±28% WB and 92%±38% WB for left and right crutches respectively. There are no significant differences involving the forces registered for two and four-point gaits. In these patterns crutches advance individually with the injured side of the body, which allows a more balanced distribution of weight, making these types of gait more stable and similar to normal walking.

As the crutch moves along the gait cycle, the pitch angle increases until it reaches its maximum angle (swing phase), at which point it starts to decrease (stance phase). For two, three-points and swing-through, the maximum values are not significant to differentiate a pattern, as shown in Table 2. However, for four points, the range of movement of the crutch is smaller than other patterns. On average these values were 14.32 deg for the left side and 13.48 for the right side. The SD remains similar for all the patterns.

In terms of accelerations, three points and swing-trough patterns showed the highest peak values. These results reflected how greater accelerations are applied in patterns that required the synchronized movement of the crutches to maintain balance. The positive peaks corresponded to the initial forward movement of the crutch, whereas the negative peaks were produced at the final instants of the crutch swing when the users stopped this forward movement. The lower acceleration peaks were recorded at two and four points, and they did not differ significantly between both patterns. The asymmetry of the crutch movement in these cases improved user balance during the gait, avoiding the need for abrupt movements when initiating the swing movement. In contrast, there is a considerable difference between the left (2.75±0.83) and right ( 2.21±0.75) accelerations (two points gait), caused precisely by this asymmetric crutch movement.

### 4.2. Crutch Gait Patterns over a Gait Cycle

**Figure 3:**
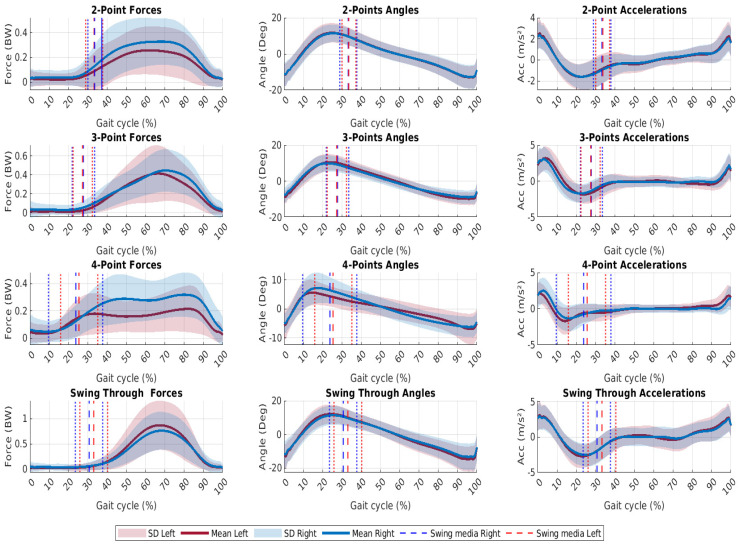
Mean forces, angle, and accelerations for all the participants in each gait pattern. The first column shows the force average, the second column represents the angles and the third column shows the accelerations. The dotted lines show the transition between the swing and stance phases.

**Figure 4:**
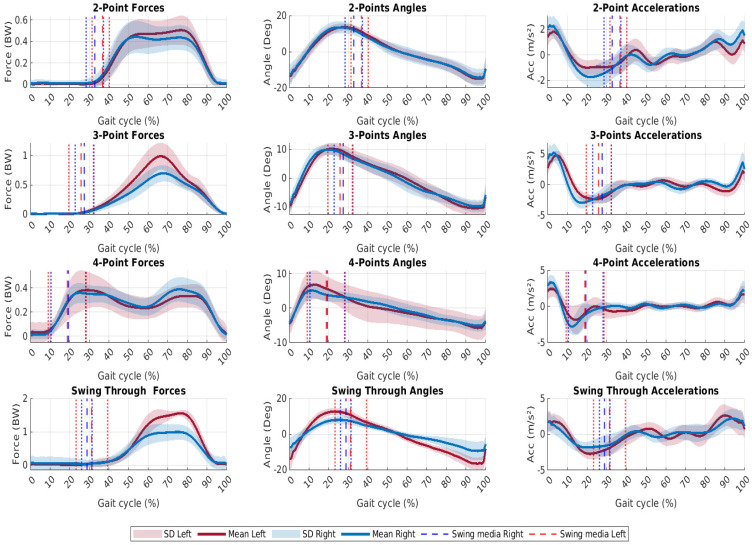
Mean forces, angle, and accelerations for Participant number 20 in each gait pattern. The first column shows the force average, the second column represents the angles and the third column shows the accelerations. Dotted lines show the range of the swing phase.

It is possible to recognize crutch gait patterns using the metrics presented in Table 2; however, this information is not sufficient to characterize a gait pattern. Graphics contained in Figure 3 show the measured signals normalized to a gait cycle for each gait pattern of all users after data processing. Meanwhile, Figure 4 shows the information for one user. It can also be observed in the first column of the graph that forces are the signals with the largest SD, as previously mentioned. In these plots, the peaks in the signal force correspond to the points when the user applied more force during a cycle. The dotted lines represent the separation between the swing and stance phases. In this paper, we call this line swing media (left and right) and it shows the end of the swing phase and the start of the stance phase. According to Figure 4, two peaks of forces can be observed in two and four-point patterns. The second peak corresponds to the transition between crutches. When comparing left and right sides, angle movements have the most graphically symmetrical shape and minor SD compared with forces. Additionally, the angle signal presents high repeatability across all patterns. In terms of symmetry and less SD, acceleration signals share similar characteristics. When the user advances with both crutches (3-points and swing-through), the accelerations are high and pattern changes are more noticeable, as seen in Figure 3.

Several results could be extracted when analyzing the graphs by gait pattern. For a two-point gait pattern, forces present two peaks, one at the start and the other one rising at the end of the stance phase, as seen in the first position of the first column in Figure 3. The second peak may be more apparent for some users when the opposite crutch moves after the dominant crutch does. This results in a greater force being applied to the dominant crutch at the end of the stance. At 25% to 35% of the gait cycle, the stance phase begins. Significant variations in force magnitude occur without exceeding the participants’ weights, which remains below 50% BW. Users performing a 3-point pattern move forward while holding both crutches and the uninjured limb. As a result, there should be just one peak along the stance phase in the distribution of forces, as shown in the graphic. The stance phase starts at 22% to 30% of the gait cycle, and maximum forces are lower than 70% BW. During a four-gait pattern, two pronounced peaking patterns are apparent. In this pattern, crutches and limbs are advanced separately. A force peak is generated when the dominant crutch touches the ground. And the second peak, when the opposite crutch is completing the cycle by striking the ground. This happens at approximately 70% to 85% of the gait cycle. During a cycle, the swing ranges from 15 to 40% and the phase stance is less than 15%. The amount of BW applied remains below 50%. In a swing-through pattern, the stance begins around 25% to 35% into the gait cycle. During most gait cycles, the peak force is greater than 100% BW. This is due to the nature of the gait pattern, which is the fastest crutch gait, but it requires more patient arm strength and balance when compared with the other patterns.

Figure 4 illustrates the patterns of the signals for user number 20. This graphic shows the points where users applied higher forces for each pattern. As an example, in the case of two and four points, the shift between crutches is evident. As shown in the force profile, the change at two points is faster and happens at 78% of the gait cycle, whereas the change at four points is slower and happens at 80% of the gait cycle. Another characteristic of this user is the high level of asymmetry in the patterns moving forward with both hands. In some cases, this occurs because the participant lacks good balance while performing the movement.

Table 3 shows the media of the stride times as well as minimum and maximum values. Differences in times are noted when comparing both crutches. However, in the three points pattern, the average stride time for both sides is similar. This time variation is minor compared with the rest of the patterns, which show more asymmetry in this parameter.

## 5. Discussion

This study primarily aimed to analyze four spatial-temporal parameters (force, acceleration, angles, and stride time) on each side, enabling the characterization of four distinct crutch gait patterns. The results imply that distinguishing between these gait patterns is possible through the examination of signal shapes and the assessment of key parameters, as demonstrated by the statistical parameters presented in this study.

The four-point pattern was observed to be the slowest and, in general, the most difficult to learn during the experiments. Contact time during the four-point pattern was longer than other crutch patterns, this characteristic was also studied in [22]. The extended contact time during this pattern may contribute to increased energy expenditure and potential discomfort for users. This insight can guide rehabilitation practitioners in tailoring interventions for individuals adopting the four-point gait, focusing on strategies to enhance efficiency and mitigate challenges associated with prolonged contact times.

Additionally, our findings based on laterality reveal that the four-point pattern, along with the two-point pattern, exhibits the most noticeable differences in force profiles. These variations may stem from the execution of these profiles involving the independent movement of the arms. Independent arm movements could indicate asymmetry or incorrect movements, emphasizing the need for rehabilitation strategies that address these aspects.

Significant SD values in the measurements are evident and can be attributed to different reasons. In general, gait patterns are individual and may be influenced by the anthropometric characteristics of the users, the training process, and the learning of movements to perform that pattern. The study faced a limitation concerning force measurement, an essential aspect given the characteristics of users, the training process, and the learning of movements required for the pattern. Additionally, variability stemming from the measurement hardware was considered, despite multiple calibrations of the sensors during the study. During the study and analysis, we did not require exact values; therefore, we considered the measurement acceptable. As a way of characterizing the pattern, we analyze percentages of weight bearing during each gait cycle. It is, however, a crucial aspect that needs to be addressed in future work. The study also included participants of different ages and physical characteristics. Seven participants applied higher forces than the average, and some participants did not correctly perform the patterns despite training. This may also influence the bias in the results. As a result of this analysis, we added a condition (condition 3) to the segmentation algorithm for two and four-point gait patterns when crutches are moved one after another.

Analysis of the symmetry was not carried out in this study. Nevertheless, the results reveal asymmetry due to comparisons between the two sides. In [23], the authors proposed a method to quantify three-dimensional GRF asymmetries in crutch-assisted walking. In that work, the participants were instructed to walk with a two-point gait with a single crutch. The results we obtained for the force signal in this pattern are comparable and are supported by this work. Also, ref. [24] proved that there is a clinical tendency to asymmetry between both steps when the non-dominant hand carried the crutch, even in healthy users. Then, our results could help the therapist to identify asymmetries and correct specific aspects of the assisted gait according to the patient.

On the other hand, the selection of parameters for gait evaluation requires experience and subjective analysis from the clinician, which may lead to investigator bias. In addition, pre-selected variables without analyzing the relationship between them and the patterns may miss potentially relevant information. For this work, gait patterns were characterized through different sets of parameters. The feature selection was made by using machine learning algorithms in our previous research. In [25] the authors applied advanced ML techniques to explore the nature of individual gait patterns in a continuous signal. In their study, they demonstrated which gait variables were most relevant for characterizing a particular individual’s gait patterns.

Using spatiotemporal parameters to describe a gait pattern objectively may enable clinicians to evaluate the quality of the individual’s gait. Knowing the contribution of each parameter to the performance of the pattern of a certain individual could support clinicians and researchers in addressing individual analysis and interventions. This paper evaluates parameters for the most common crutch gait patterns. Prior studies have shown the importance of understanding the interaction between crutches and users. Our results provide a means to observe how most common patterns are performed by assessing a typical set of metrics (forces, accelerations and angles).

For some exoskeletons, the balance is achieved by the user through the use of crutches and leg motion, as shown in [26]. Instrumented crutches could be part of the exoskeleton control system to personalize the controllers and assist users by detecting their walking patterns and user intentions.

Finally, We chose to study healthy participants for several reasons. First, they provide a baseline for normal crutch gait patterns, aiding in the identification of deviations in patients. Second, studying healthy individuals allows us to isolate the influence of the crutch technique without the interference of variables due to medical conditions. This approach enhances methodological rigor, duplicability, and the systematic exploration of spatial-temporal parameters. Additionally, starting with healthy participants addresses ethical considerations associated with experiments involving patients. While our study represents an initial stage, future research will extend to patient populations, ensuring a comprehensive understanding of crutch gait patterns and their implications for rehabilitation.

## 6. Conclusions

In this study, we present findings from the utilization of instrumented crutches to evaluate and characterize four distinct crutch walking patterns through the analysis of spatial and temporal parameters. To achieve this, 27 healthy participants engaged in multiple trials featuring the execution of four different gait patterns. A portable system integrated within the crutches facilitated the measurement of axial forces, linear accelerations along the x, y, and z axes, as well as the crutches’ orientation angles during each trial. An algorithm utilizing pitch angle data was developed to segment trials into phases and discern these phases through a heuristic approach. Through the measurement of forces, accelerations, pitch angles, and stride times, we successfully delineated and described walking patterns associated with assisted crutch ambulation. In contrast to our previous works that focused on developing prototypes and selecting metrics for analysis, this study delves deeper into the examined patterns. We expanded our experiments to include a significant number of individuals to validate our hypotheses. Upon comparison of the parameters among the 27 participants across different crutch gait patterns, notable similarities and differences emerged. The selected spatial and temporal parameters yielded promising outcomes for the monitoring and evaluation of assisted gait with crutches. Particularly, graphical representations of the patterns facilitated the comprehension of their dynamics and facilitated the extraction of pertinent values on an individual basis (such as maximum/minimum forces, accelerations, and angles). Furthermore, a significant finding of this study suggests that instrumented crutches hold potential as a valuable clinical tool for enhancing diagnosis, assessment, training, and treatment through the monitoring and analysis of gait execution.

## Figures and Tables

**Table 1:** Features summary.

Type	Features
Temporal	Stride Time, swing and stance time
Statistical Features	Mean, Std, Min and Max
	Acc.X, Acc.Y, Acc.Z, Acc. Module, Roll, pitch, yaw and Force
Motion-based	%Swing phase, %Stance phase, Number of stance events, number of swing events, force ration, Swing ratio, Stance ratio

**Table 2:** Descriptive statistics of the collected measures (Force, Angle, Acceleration) for both crutches within a gait pattern. Force values are normalized to the user’s BW.

Gait Type	Side	Measurement	Minimum (SD)	Maximum (SD)
two points	Left	Force (BW)	0.03±0.02	0.37±0.18
		Angle (°)	−13.31±2.13	11.94±1.59
		Acceleration ($m/s^{2}$)	−1.80±0.46	2.43±0.56
	Right	Force (BW)	0.02±0.01	0.31±0.17
		Angle (°)	−13.93±2.46	12.55±2.37
		Acceleration ($m/s^{2}$)	−1.77±0.40	2.53±0.58
three points	Left	Force (BW)	0.02±0.02	0.49±0.19
		Angle (°)	−9.16±1.51	10.54±2.32
		Acceleration ($m/s^{2}$)	−1.97±0.62	3.41±0.92
	Right	Force (BW)	0.01±0.01	0.46±0.25
		Angle (°)	−10.07±1.64	11.11±2.31
		Acceleration ($m/s^{2}$)	−2.04±0.66	3.51±1.00
four points	Left	Force (BW)	0.04±0.03	0.37±0.15
		Angle (°)	−6.41±1.52	7.91±2.23
		Acceleration ($m/s^{2}$)	−1.67±0.78	2.75±0.83
	Right	Force (BW)	0.02±0.01	0.32±0.13
		Angle (°)	−7.42±2.64	6.06±2.07
		Acceleration ($m/s^{2}$)	−1.89±0.78	2.21±0.75
Swing-Through	Left	Force (BW)	0.02±0.02	0.83±0.28
		Angle (°)	−14.73±4.57	12.10±2.99
		Acceleration ($m/s^{2}$)	−2.68±0.62	3.12±0.65
	Right	Force (BW)	0.01±0.01	0.92±0.38
		Angle (°)	−15.87±4.09	12.58±2.32
		Acceleration ($m/s^{2}$)	−2.86±0.68	3.43±0.77

**Table 3:** Stride time descriptive statistics for both crutches. Mean and SD values, units in seconds.

Stride Time					
Side Crutch		Left		Right	
Gait Type	Parameter	Mean	Min/Max	Mean	Min/Max
Two points	Stride	1.79±0.18	1.56/2.06	1.83±0.21	1.55/2.09
Three points	Stride	2.05±0.2	1.35/2.85	2.06±0.16	1.65/2.5
Four points	Stride	2.53±0.48	1.65/4.2	2.41±0.39	1.06/3.26
Swing-Through	Stride	1.59±0.36	1/2.7	1.66±0.44	1.15/3.2

## Data Availability

Data are contained within the article.

## Appendix A

Table A1 shows the age, size and weight of the participants in the experiments.

**Table A1:** Subjects data.

Subject ID	Age	Height (cm)	Weight (kg)
1	23	192	86
2	40	177	96
3	33	167	95
4	26	185	83
5	24	162	72
6	23	180	60
7	66	162	67
8	25	165	51
9	31	168	86
10	24	160	56
11	25	170	63
12	28	166	50
13	24	181	75
14	24	186	77
15	34	176	82
16	33	174	54
17	23	165	66
18	24	170	72
19	30	175	63
20	28	164	65
Mean	29.40	172.25	70.95
Std	9.80	9.04	13.86
